# A genetic polymorphism evolving in parallel in two cell compartments and in two clades

**DOI:** 10.1186/1471-2148-13-9

**Published:** 2013-01-12

**Authors:** Ward B Watt, Richard R Hudson, Baiqing Wang, Eddie Wang

**Affiliations:** 1 Department of Biology, Stanford University, Stanford, CA 94305-5020, USA; 2 Rocky Mountain Biological Laboratory, Crested Butte, CO 81224, USA; 3 Department of Ecology and Evolution, University of Chicago, Chicago, IL, 60637, USA; 4 Present address: Department of Molecular and Cellular Biology, Harvard University, Cambridge, MA, 0213, USA

**Keywords:** Amino acid polymorphism, Coalescent simulation, Glycolysis, Intramolecular bond variation, Neutral null hypothesis, Parallel evolution, Phosphoenolpyruvate carboxykinase, Selection hypothesis, Splice variation

## Abstract

**Background:**

The enzyme phosphoenolpyruvate carboxykinase, PEPCK, occurs in its guanosine-nucleotide-using form in animals and a few prokaryotes. We study its natural genetic variation in *Colias* (Lepidoptera, Pieridae). PEPCK offers a route, alternative to pyruvate kinase, for carbon skeletons to move between cytosolic glycolysis and mitochondrial Krebs cycle reactions.

**Results:**

PEPCK is expressed in both cytosol and mitochondrion, but differently in diverse animal clades. In vertebrates and independently in *Drosophila*, compartment-specific paralogous genes occur. In a contrasting expression strategy, compartment-specific PEPCKs of *Colias* and of the silkmoth, *Bombyx*, differ only in their first, 5^′^, exons; these are alternatively spliced onto a common series of following exons. In two *Colias* species from distinct clades, PEPCK sequence is highly variable at nonsynonymous and synonymous sites, mainly in its common exons. Three major amino acid polymorphisms, Gly 335 ↔ Ser, Asp 503 ↔ Glu, and Ile 629 ↔ Val occur in both species, and in the first two cases are similar in frequency between species. Homology-based structural modelling shows that the variants can alter hydrogen bonding, salt bridging, or van der Waals interactions of amino acid side chains, locally or at one another^′^s sites which are distant in PEPCK^′^s structure, and thus may affect its enzyme function. We ask, using coalescent simulations, if these polymorphisms^′^ cross-species similarities are compatible with neutral evolution by genetic drift, but find the probability of this null hypothesis is 0.001 ≤ P ≤ 0.006 under differing scenarios.

**Conclusion:**

Our results make the null hypothesis of neutrality of these PEPCK polymorphisms quite unlikely, but support an alternative hypothesis that they are maintained by natural selection in parallel in the two species. This alternative can now be justifiably tested further *via* studies of PEPCK genotypes^′^ effects on function, organismal performance, and fitness. This case emphasizes the importance, for evolutionary insight, of studying gene-specific mechanisms affected by natural genetic variation as an essential complement to surveys of such variation.

## Background

**Figure 1:**
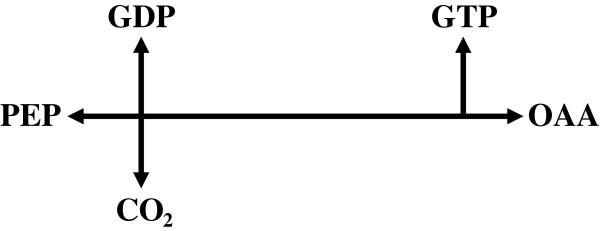
**Reaction catalyzed by PEPCK.** Abbreviations: PEP, phosphoenolpyruvate; GDP and GTP, guanosine di- and tri-phosphate; CO_2_, carbon dioxide; OAA, oxaloacetate.

Phosphoenolpyruvate carboxykinase, PEPCK, converts phosphoenolpyruvate (PEP) plus nucleotide diphosphate and carbon dioxide to and from oxaloacetic acid (OAA) plus nucleotide triphosphate, in multiple metabolic contexts among the domains of life. Its guanosine-nucleotide-using form (EC 4.1.1.32; for reaction see Figure 1), while present in some Bacteria and Archaea, occurs mainly in Animalia and in both cytosol and mitochondrial matrix. Its production of PEP from OAA begins gluconeogenesis or glycerol synthesis from Krebs cycle metabolites, or through them from dietary amino acids or lipids [1]. Its production of OAA from PEP may ″replenish″ Krebs cycle metabolites, or play a role in reaction paths which produce moderate ATP yields during chronic anoxia in some invertebrates [2]. In mice, its overexpression in skeletal muscle yields striking extensions of exercise capacity, lifespan, and reproduction [3].

We have studied natural genetic variation in enzymes of energy metabolism, using *Colias* (Lepidoptera, Pieridae) as a test system for evolutionary functional genomics [4,5]. While surveying such variation across all enzyme-coding genes of glycolysis and its links to other processes, separate study of PEPCK was prompted by finding 3 high-frequency amino acid polymorphisms at the same PEPCK codons in two *Colias* species from distinct clades. We first clarify the basis of dual cell compartment expression of PEPCK in *Colias vs.* other animals. We next study PEPCK^′^s natural variation in the two *Colias* species, especially the high-frequency amino acid variants shared between species. We locate these variants in PEPCK^′^s structure and explore their possible effects on structure-function relations. With coalescent simulations, we test a population-genetic null hypothesis of genetic drift as cause of this variation, the alternative cause being natural selection.

## Methods

### Animals and basic molecular biology

PEPCK cDNA was made by reverse-transcription (with Invitrogen MMLV enzyme) of mRNA extracted from fat body (preserved in Ambion RNAlater) of 18 *Colias eurytheme* (randomly sampled near Tracy, California, elevation 25 m) and 18 *Colias meadii* (randomly sampled from Cottonwood Pass, Colorado, elevation 3780 m). Using ButterflyBase [6], we compared PEPCK sequences from *Bombyx mori* (BMP026541_1) and *Heliconius erato* (HEP05212_1) to design consensus primers for initial PCR amplification (using Invitrogen HiFi Platinum Taq and Stratagene Robo-cyclers) of a central sequence fragment of *Colias* PEPCK cDNA. Primers matching this fragment were designed, using Oligo 6 software (Molecular Biology Insights, Inc.), first to amplify the gene^′^s 3^′^ end with an antisense primer matching the cDNA polyA tail (GEN22(15)-A3end: [4,5]), and then, using the Ambion RLM-RACE kit, to amplify the 5^′^end (unexpectedly complex, as discussed below). *Colias*-specific primers were then designed (Additional file 1) to amplify and sequence the whole gene from 5^′^ to 3^′^ untranslated regions (UTRs). Nucleic acids were purified with Qiagen kits and a Qiacube processing robot. Sequences were read in sense and antisense directions with ABI BigDye 3.1 reagents and an ABI 377 sequencer.

#### Data processing and bioinformatics

*Colias* PEPCK sequences were cross-checked and edited using BioEdit [7]. DnaSP 5.1 [8] was used to estimate haplotype compositions from individuals^′^ heterozygous PEPCK sequences using the PHASE algorithm [9,10], and to tabulate diverse evolutionary-genetic statistics from these sequences; previously written filter programs [5] were used to organize DnaSP analysis of linkage disequilibrium. Sequences of *Bombyx mori*^′^*s* PEPCK were retrieved from expressed sequence tag libraries in ButterflyBase [6] and from assembled genomic DNA in SilkDB 2.0 [11], using search tools of each site. *Drosophila* sequences were drawn from FlyBase [12], and vertebrate and prokaryotic sequences from GenBank [13].

All sequences were evaluated for cell compartment specificity using the TargetP server [14]. This server^′^s elaborate algorithm assesses mitochondrial targeting of proteins, as contrasted to properties of proteins retained by default in the cytosol, on the basis of characteristics of their N-terminal amino acid sequences: a) richness in basic (Arg, Lys) and hydroxylated (Ser, Thr) amino acids; b) absence of acidic amino acids (Asp, Glu); c) certain secondary structure features [14].

Sequences were aligned with the ClustalW algorithm as implemented in BioEdit. Additional file 2 lists accession numbers of all sequences, including those from *Colias* as submitted to GenBank. Phylogenetic relationships among sequences were evaluated with PHYML and PHYLIP software [15,16]. *Colias* sequences were matched to the best available structural templates, for homology-based structural modelling, by the 3D-Jury metaserver [17]. Template structure files were drawn from the Protein Data Bank [18]. Homology-based calculation of *Colias* PEPCK structures using these templates was done with MODELLER 9.8 [19]; in each case, 5 replicates were run and the best-scoring one (i.e. with lowest value of the molpdf criterion [19]) was used. These models were visualized, and their structural features measured, with DeepView (Swiss-PDB Viewer) 4.0.1 [20].

## Results

### Basic genomic structure of PEPCK in Colias, other insects, and vertebrates

**Figure 2:**
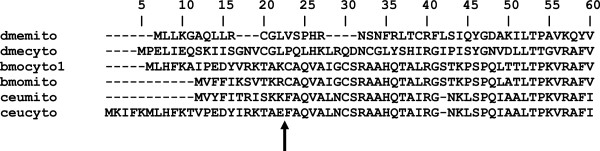
**5**^′^**ends of insect PEPCK amino acid sequences.** Abbreviations: dme, *Drosophila melanogaster*; bmo, *Bombyx mori*; ceu, *Colias eurytheme*; cyto, cytosol form; mito, mitochondrial form. Arrow marks the splice break between the alternative 5^′^ exons 1 and the common block exons 2–13 in *Bombyx* and *Colias*. The numbering of this figure does not match the standard numbering (based on the mito form) of *Colias* base pair and amino acid positions used in the rest of the paper: e.g., the splice break occurs between amino acids 11 and 12 in the standard numbering.

During primer set development for the autosomal *Colias* PEPCK gene, we found two sequence forms, differing in their 5^′^ ends and 5^′^-untranslated regions (UTRs). Inspection of *Bombyx mori*^′^*s* PEPCK sequences [6] clarified this: PEPCK sequence BMP026541_1, closely matching one of the *Colias* forms, is annotated to cytosolic expression, and BMP000643_1, close in sequence to the other *Colias* form, to mitochondrial expression. The TargetP server confirmed this compartment targeting for the two forms in each species. In each taxon, these sequences differ only in their 5^′^ ends, being mRNA splice variants whose alternative 5^′^ exons (each associated with a unique 5^′^ untranslated region, in which unique 5^′^ amplifying primers are located) are attached to common exons 2–13. (That the sequences following the 5^′^ exon are the same, and not parts of fully distinct paralogs, was shown in *Colias* by the fact that in every case, in amplifying from the 5^′^ untranslated region, regardless of which of the two 5^′^ exons was amplified, all varying base positions following the 5^′^ exon, whether heterozygous or variant-homozygous in an individual, were the same between the alternately amplified sequences.) But in *Drosophila melanogaster,* distinct, though closely linked, paralogous genes code for PEPCK of cytosol and of mitochondria (12; Figure 2 shows these insects^′^ PEPCK 5^′^ ends). Pairs of compartment-specific paralogs also occur in diverse vertebrates [13].

A truncated expressed-sequence-tag sequence from *Bombyx*, similar but not identical to BMP026541_1, occurs in ButterflyBase as BMP026778_1. Consultation of the *Bombyx* genome assembly [11] clarified this. The whole gene corresponding to BMP026541_1, including the cytosolic exon 1, occupies one locus, punctuated by 12 introns, on the minus strand of scaffold nscaf2789. Roughly 20 kb beyond this on the minus strand begins the second locus BMP026778_1, which is interrupted by base dropouts causing frameshifts in comparison to BMP026541_1; if the first of these is ″repaired″ by substitution from BMP026541_1, more PEPCK-like sequence is recovered to about codon 235, after which more frame-shifting results in premature stop codons. Thus this locus behaves like an incipient, but not yet fully silenced, pseudogene. We′ve found no expressed mRNA sequence evidence of any such locus in *Colias*.

*Bombyx*′ mitochondrial exon 1 of BMP000643_1, with its distinctive 5^′^ -untranslated region, is again on the minus strand, 15 kb beyond BMP026778_1. It is not now annotated in SilkDB, so we give its specific location here: nscaf 2789: exon bp 1207835 – 1207867, 5^′^-UTR to ~ 1207900.

### Evolutionary history of PEPCK

**Figure 3:**
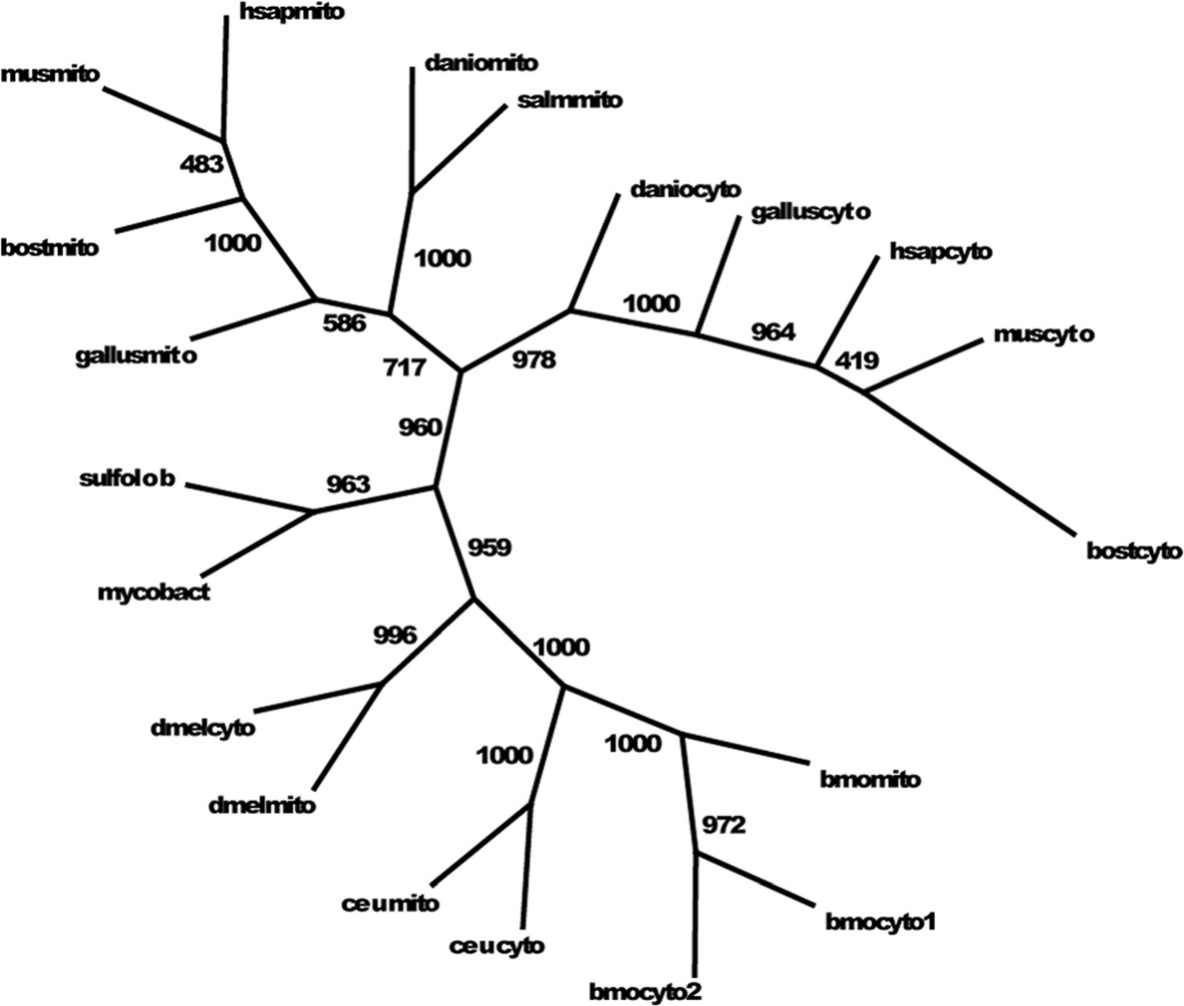
**Maximum likelihood reconstruction of PEPCK gene phylogeny.** Numbers at edges are bootstrap support values from 1000 iterations using a JTT amino acid substitution model in PHYML [15]. Sequences were bootstrapped and iterations compiled with SEQBOOT and CONSENSE from the PHYLIP package [16]. Abbreviations are those of Figure 2 plus: mycobact, *Mycobacterium sp.* (Eubacteria); sulfolob, *Sulfolobus sp.* (Archaea); danio, *Danio rerio* (zebrafish); salm, *Salmo salar* (salmon); gallus, *Gallus gallus* (chicken); bost, *Bos taurus* (cattle); mus, *Mus domesticus* (housemouse); hsap, *Homo sapiens* (human.

What is the evolutionary history of the alternate PEPCK expression strategies – paralogs *vs.* splice variants? One study has examined the phylogenetic relationships of the GTP-using (E.C. 4.1.1.32) and ATP-using (E.C. 4.1.1.49) PEPCK enzymes [21]. It focused on distribution of these co-substrate types among domains Bacteria, Archaea, and Eukarya, but did not address the eukaryotic cell-compartment-specific forms. Therefore we reconstructed, using protein sequences, the phylogeny of vertebrate and insect GTP-using PEPCKs, with prokaryotic GTP-using PEPCKs as outgroups, drawing sequences from sources listed above. Figure 3 shows that vertebrate mitochondrial and cytosolic PEPCKs form two compartment-specific paralogous branches whose most basal members on each branch are fish sequences. This apparent duplication-and-divergence event may have been part of the whole-genome duplications found at the base of vertebrate evolution [22]. The *Drosophila* paralogs form a coherent sub-branch of an Insecta branch, originated independently of the vertebrate paralogs, evolving the duplication-and-divergence genomic mechanism for compartment-specificity in parallel. The apparent *Bombyx* pseudogene (bmocyto2; BMP026778_1) groups closely with the main *Bombyx* cytosolic gene; it is unrelated to the *Drosophila* paralog pair. The exon-splicing mechanism of the two Lepidoptera, *Bombyx* and *Colias*, is a distinct alternative to whole gene paralogy for compartment-specificity of PEPCK.

### Overall sequence variation of Colias PEPCK

The cDNA of *Colias* cytosolic PEPCK includes 1929 bp, 643 codons, and the mitochondrial form includes 1896 bp, 632 codons (omitting the stop in each case). The length differences lie in the 5^′^ exons of 22 and 11 codons respectively; base pair and amino acid sites for the gene as a whole are numbered beginning with the mitochondrial 5^′^ exon. Cytosol form sequences add 11 codons/33 base pairs (bp) to numbers beyond the end of the cytosol exon 1. The mitochondrion-targeting exon 1 may be excised once its protein has been imported into mitochondria, as this happens with other such proteins, ostensibly to prevent further interaction with the mitochondrial membranes [23]. If so, this would affect interpretation of both structural and population-genetic aspects of mitochondrial PEPCK′s functional evolutionary interactions (see below).

18 *C. eurytheme* and 18 *C. meadii* were sequenced for both 5^′^ exons and the common exons 2–13, and haplotype phases were estimated for each species as noted above. Genetic statistics are tabulated in Table 1 for the common exons 2–13 and for the two 5^′^ exons. PEPCK is highly variable at both amino acid and DNA levels: e.g., for exons 2–13 of *Colias eurytheme*, in common between the compartment forms, overall nucleotide diversity π = 0.0269, synonymous diversity π_ss_ = 0.1054, and θ = 4N_e_μ = 0.028 (estimated from the number of segregating sites S). Both 5^′^ exons are less variable than exons 2–13. In comparison, *Colias eurytheme* PGI, one of the most variable animal genes known (maintained so by strong natural selection [4]), shows π_Σ_ = 0.0267, π_ss_ = 0.0993, and θ = 0.034 [5], while average values for a sample of *Drosophila melanogaster* genes are π_Σ_ = 0.0040, π_ss_ = 0.0135, and θ = 0.0040 [24]. *Colias*^′^ PEPCK thus matches its PGI in level of variability. It also shows similarly high estimates of the minimum number of intragenic recombination events [25], i.e. 60 and 41 for *C. eurytheme* and *C. meadii* respectively, *vs.* 58 in the 1668 bp of *C. eurytheme* PGI [5].

**Table 1:** **Genetic statistics of *Colias *PEPCK**

**Taxon and form**	**n**	**subset**	**bp**	**S**	**#var**	**π**	**k**	**θ**	**min recs**
*C. eurytheme*									
Common exons 2-13	36	Σ	1863	220	255	0.0269	50.06	0.028	60
		nss	1424	27	28	0.0027	3.80		
		ss	439	193	227	0.1054	46.25		
cyto 5^′^ exon 1	36	Σ	66	3	3	0.0143	0.94	0.011	
		nss	54	1	1	0.0010	0.06		
		ss	12	2	2	0.0739	0.89		
mito 5^′^ exon 1	36	Σ	33	1	1	0.0154	0.58	0.015	
		nss	26	1	1	0.0197	0.51		
		ss	7	0	0	0.0000	0.00		
*C. meadii*									
Common exons 2-13	36	Σ	1863	158	173	0.0209	38.89	0.020	41
		nss	1424	21	21	0.0020	2.89		
		ss	439	137	152	0.0821	36.02		
cyto 5^′^ exon 1	36	Σ	66	1	1	0.0037	0.25	0.004	
		nss	54	0	0	0.0000	0.00		
		ss	12	1	1	0.0021	0.25		
mito 5^′^ exon 1	36	Σ	33	0	0				
		nss	26	0	0				
		ss	7	0	0				

Tabulations done with DnaSP 5.1. Abbreviations: *n*, total number of alleles sequenced; subset, classes of sites within cDNA; *bp*, base pairs (per subset); *Σ*, all substitution sites; ss, synonymous sites; *nss*, nonsynonymous sites; *S*, number of segregating sites; *#var*, number of variants; *π*, nucleotide diversity per bp; *k*, average difference of nucleotides, pairwise among sequences (per whole gene); *θ, 4N*_*e*_*μ,* estimated from S, where N_e_ is effective population size and μ is mutation rate; min recs, minimum recombinations among sequences, estimated by the four-gamete test [25]; *cyto*, cytosol; mito, mitochondrial. Mean numbers of pairwise nucleotide differences between species for whole mito form: all sites 52.50, ss sites 47.49, nss sites 5.02. No sites are fixed for different substitutions between the two species.

### Patterns of allelic amino acid variation

**Figure 4:**
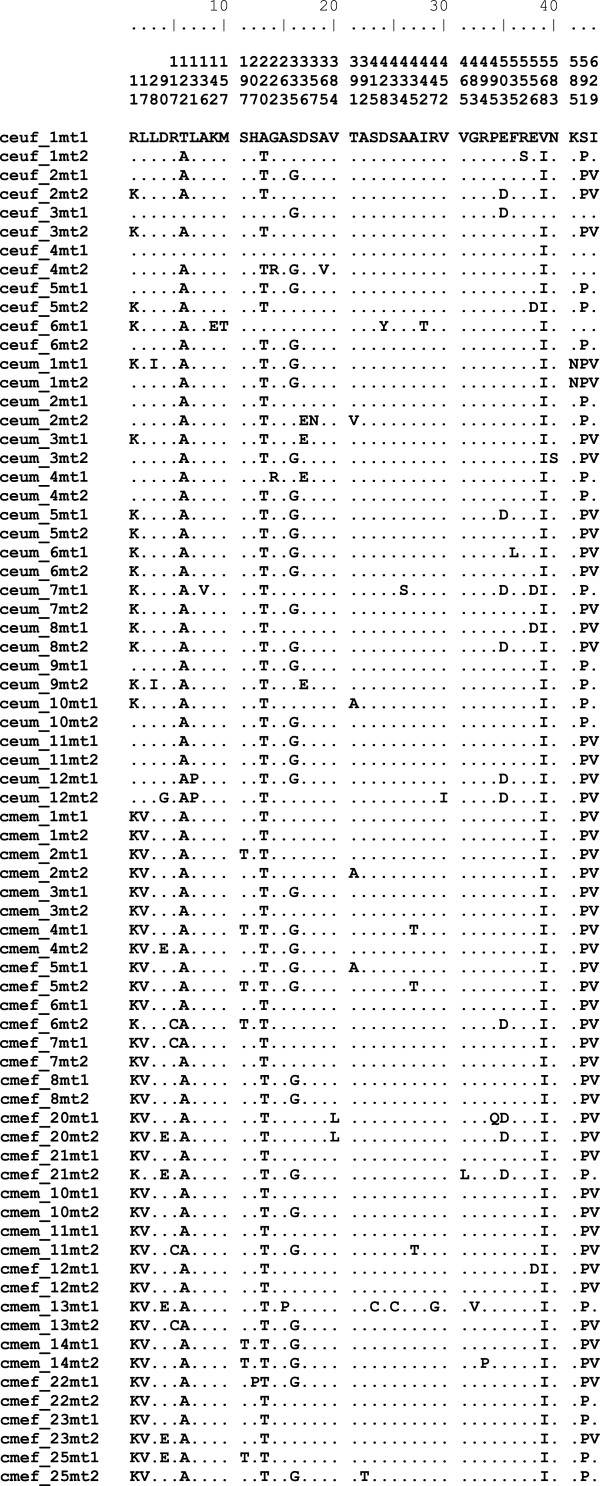
**Amino acid sequence variation of *Colias *PEPCK (as mitochondrial form).** Sequence names are formed from species abbreviations as in Figure 2, “m” or “f” for specimen sex, specimen numbers, and “mt1” and “mt2” for alleles inferred from heterozygous sequences with the PHASE algorithm as discussed in the text. “.” denotes identity with the first sequence listed. The scale at the top of the figure counts the variant positions, while the vertically formatted numbers below it identify the codons‘placement in PEPCK’s amino acid sequence.

Each unique PEPCK sequence at either nucleotide or amino acid level of organization constitutes a distinct genetic allele at that level. We focus here on amino acid variation as the possible basis of naturally selected enzyme properties, thence effects on higher-level phenotypes and eventually on Darwinian fitness. Figure 4 shows all mitochondrial-form amino acid allelic variants as inferred by PHASE (above). Nearly all the variation occurs in those exons, 2–13, which are in common between the compartment forms. 23 codons have singleton amino acid variants in *C. eurytheme* or *C. meadii* while 20 codons have major polymorphism (p_2_ ≥ 0.05) in either species (the cytosol 5^′^ exons have only one singleton in *C. eurytheme*, none in *C. meadii*). These combine into 28 (of 36) distinct alleles in *C. eurytheme* and 24 (of 36) distinct alleles in *C. meadii*.

Three polymorphic codons, 335, 503, and 629, have p_2_ ≥ 0.05 in both species: Gly/Ser 335, Asp/Glu 503, and Ile/Val 629. All these changes are charge-neutral. By exact binomial test [26], corrected for multiple tests [27], codon 629 differs significantly in its variant frequencies between species, while codons 335 and 503 do not (Table 2). These variants combine into 8 allele classes or allele ″macrostates″ [5], which are identified by one-letter amino acid codes at each position, e.g. GEI for Gly Glu Ile). Frequencies of these alleles for the two species are given in Table 3; their differences mostly follow *C. eurytheme*^′^s increase in Ile 629 frequency compared to *C. meadii*.

**Table 2:** **Major PEPCK amino acid variation shared by *Colias *species**

**Taxon**				**Codons**					
		**335**			**503**			**629**	
	**n**	***p***	**aa**	**n**	***p***	**aa**	**n**	***p***	**aa**
CEU	20	0.56	Gly	7	0.19	Asp	18	0.5	Ile
	16	0.44	Ser	29	0.81	Glu	18	0.5	Val
CME	15	0.42	Gly	4	0.11	Asp	6	0.17	Ile
	21	0.58	Ser	32	0.89	Glu	30	0.83	Val
x*		1.18			0.98		−3.00		
P		0.24			0.33		0.003		

*CEU*, *C. eurytheme*; *CME*, *C. meadii*; *n*, variant counts; *p*, variant frequencies; *aa*, amino acids; x*, test parameter (normal deviate) for Goldstein’s exact binomial test for frequency difference [26]; *P*, probability of as much or more difference between samples by chance. Given n = 3 codons to test simultaneously, the corrected (Dunn-Sidak) minimum significance level α′ = [1 – (1– α)^1/n^ = 0.017 [27].

**Table 3:** **Frequencies of 8 PEPCK allele classes sampled from two *Colias *species**

	***C. eurytheme***		***C. meadii***	
**Allele**	**Count**	**Frequency**	**Count**	**Frequency**
GDV	3	0.083	0	0.000
GDI	1	0.028	1	0.028
GEV	10	0.278	13	0.361
GEI	6	0.167	1	0.028
SDV	2	0.056	3	0.083
SDI	1	0.028	0	0.000
SEV	3	0.083	14	0.389
SEI	10	0.278	4	0.111
All	36		36	

Allele names, based on the 3 shared polymorphic amino acid sites, are composed of standard one-letter symbols for amino acids segregating at codons 335, 503, and 629 (see the text).

We asked if there is intragenic linkage disequilibrium among codon 335, 503, and 629 variants; as Additional file 3 shows, there is not. For reasons noted below, we also tested *C. eurytheme* for disequilibrium between these 3 codons and the 5^′^-mitochondrial-exon codon 11 Arg/Lys polymorphism of *C. eurytheme*, but none was found (Additional file 3). Indeed, scanning whole cDNA sequences of each species for linkage disequilibrium with DnaSP and filters (Methods, above) found only one instance of disequilbrium between nonsynonymous variants (codons 122 and 220 gave a locally significant Fisher^′^s exact test at P = 0.001, but this was not significant by DnaSP′s Bonferroni criterion for multiple testing) in *C. eurytheme*, and no such instances in *C. meadii*. Absence of disequilibria among amino acid variants fits with the finding above of extensive intragenic recombination in PEPCK of both species. Some mainly nearby disequilibria involving synonymous variants are seen, but no interpretation is evident and we omit these data for the sake of brevity.

On a hypothesis of selective neutrality some variable sites are expected to be shared between species, given large θ and thus a number of sites variable in each species at a time [28]. But on this hypothesis, the vast majority of those variants are expected to be of very low frequency and destined to be lost by drift to fixation [29]. A finding of multiple high-frequency variants shared between species at even roughly similar frequencies is unusual on this hypothesis, and so merits study of its possible causes – neutrality as null hypothesis, or some form of natural selection as an alternative. Direct study of the variants^′^ functional effects, and fitness consequences in the wild, will be the future and final arbiter of this issue. But we can even now make more use of present data, by homology-based structural modelling and by population-genetic simulation, to gain further insight.

### Structural nature and potential impacts of amino acid sequence variation

We summarize PEPCK^′^s protein structure in order to study placement of its polymorphic variants in *Colias* for clues to their evolutionary meaning. This is a first step in assessing neutrality or selection in functional terms: if molecular modelling shows changes in intramolecular protein bonding by amino acid variants, that may at least suggest variants^′^ possible functional effects, while if no such changes are evident, functional neutrality of the variants is strongly suggested.

PEPCK is one of a few enzymes of glycolysis and related processes which are active as monomers, without oligomeric structure. The best homologous modelling template available, *per* 3D-Jury [17], is PEPCK of *Rattus*: high-resolution crystal structures exist for two catalytic conformations of this ([30,31], see Additional file 4 for a *ColiasRattus* alignment). Sequence identity between *Colias* and *Rattus* PEPCKs is 0.61, and their Dayhoff similarity is 0.77. These values support homology-based modelling, with accuracy of mid-range crystallographic resolution [19], to explore variants^′^ potential structural effects. We modelled both conformations of each amino acid polymorph allele for each of three compartment-specific forms: cytosolic exon 1 plus common exons 2–13, mitochondrial exon 1 plus exons 2–13, and exons 2–13 alone in light of the above-noted likelihood that mitochondrial exon 1 is excized once mitochondrial PEPCK has reached its target.

**Figure 5:**
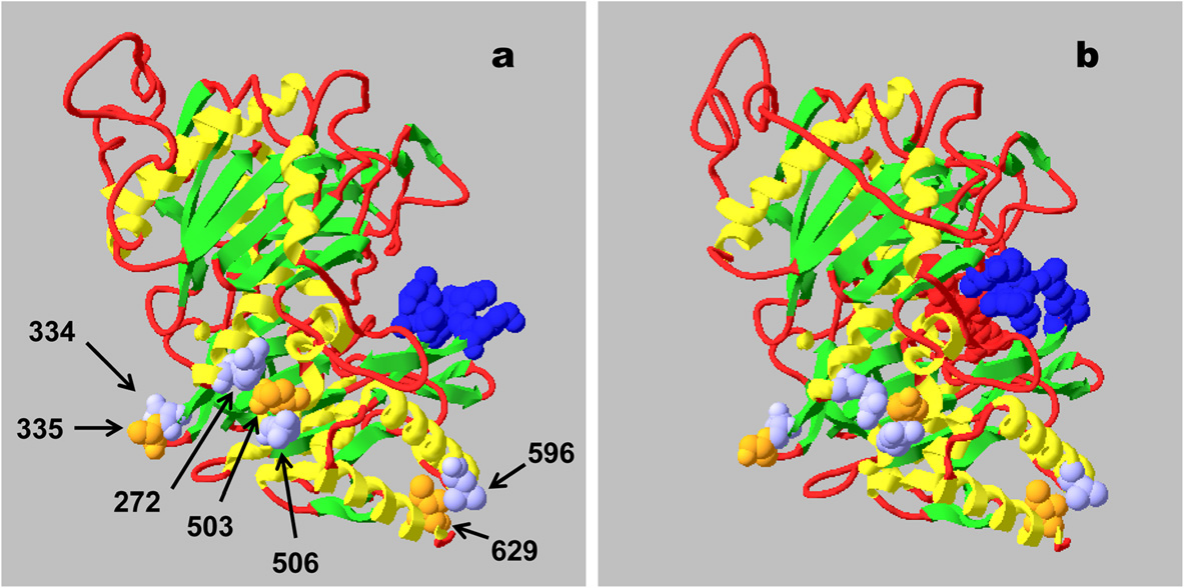
**Homology-based models of a) “lid-open” and b) “lid-closed” conformations of *Colias *PEPCK.** Models represent the cytosol form of allele Ser 335 – Glu 503 – Ile 629 (SEI). Ribbon model color code: green, β-strands; yellow, α-helices; red, loops. Space-filling model color code: red, substrates bound into catalytic center in b); dark blue, mobile lid loop; orange, polymorphic amino acid sites 335, 503, and 629 (standard numbering) which are shared between species; light blue, invariant amino acid sites Arg 272, Asp 334, Arg 506, and Leu 596. Note changes from structure **a**) with lid closure in structure **b**): separation of side chains of Asp 334 and Ser 335 with loss of hydrogen bond (cf. Figure 6); movement of Glu 503 to the right, and Arg 272 down, relative to Arg 506, forming 503–506 salt bridge (cf. Figure 7); loss of strong van der Waals contact between Leu 596 and Ile 629; shortening of the left end of the α-helix containing Leu 596.

**Figure 6:**
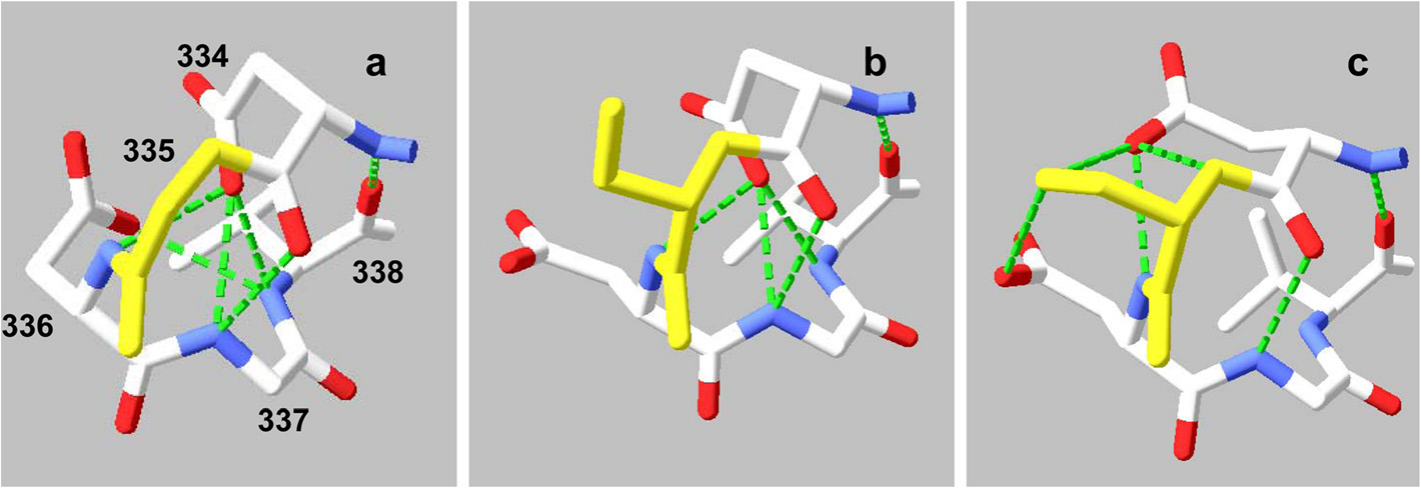
**Hydrogen bond alternatives for PEPCK polymorphic site Gly/Ser 335.** Models are of the cytosol form in lid-open conformation**.** Color codes: polymorphic amino acid yellow, others’ carbon skeletons white with oxygens red and nitrogens blue. Hydrogen bonds between Asp 334 backbone nitrogen and Val 338 carbonyl, Asp 334 carboxyl and Asp 336 backbone nitrogen, and Asp 334 carbonyl and Gly 337 backbone nitrogen are invariant, while others change with alleles. **a**) allele GEV, Gly 335 with no hydrogen bonds; **b**) allele SEV, Ser 335 with no hydrogen bonds; **c**) allele SEI, Ser 335 hydrogen bonding between its backbone nitrogen and Asp 334’s carboxyl, and also hydrogen bonding between its sidechain hydroxyl and the carboxyls of either Asp 334 or Asp 336. These models do not prioritize among alternative potential hydrogen bonds in case of multiple such bonds per atom.

Figures 5a,b show that *Colias* PEPCK^′^s tertiary structure is built up from its secondary structure as an irregular lattice of β-strands. This supports α-helices which form much of the protein^′^s surfaces. Ends of these α- and β-structures are connected by loops. The catalytic center includes a mobile ″lid″ loop which when open (structure PDB 2qew, Figure 5a) allows substrate/product binding or release, but closes (structure PDB 2qf2, Figure 5b) over these ligands when they are bound during catalysis [30-32]. Other kinds of changes accompany this lid movement, e.g.:

• the ″p-loop″, including substrate-binding residues Cys 304 and Lys 306, moves in the catalytic site with the lid′s movement [30,32];

• amino acids 592–610 form an α-helix when the lid is open, but the helix appears to shorten to 592–609 when the lid is closed (see Figures 5a,b);

• invariant and polymorphic amino acids can change spatial relations and resulting bond patterns when the lid is open *vs.* closed, as seen in Figure 5 and supplemented by Figure 6.

**Figure 7:**
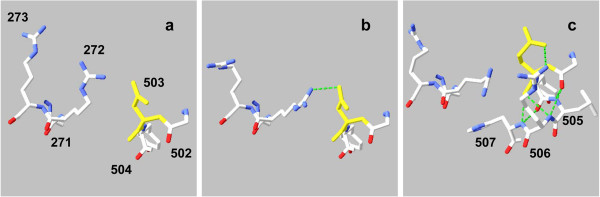
**Salt bridge alternatives for PEPCK polymorphic site Asp/Glu 503.** Models are of the cytosol form in lid-closed conformation. Color codes as for Figure 6. **a**) allele SDI, no salt bridge; **b**) allele SDV, salt bridge between the carboxyl of Asp 503 and a nitrogen of the guanidino side chain of nearby Arg 272; **c**), allele SEI, salt bridge between Glu 503 carboxyl and the guanidino side chain of Arg 506, near Asp/Glu 503 in the same α-helix, as shown in coarser scale by Figure 5b. Arg 272's side chain can sometimes hydrogen-bond to the backbone carbonyl of Glu 503 (not shown).

**Figure 8:**
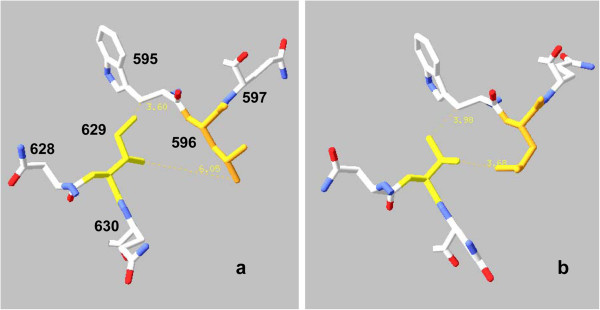
**Absence or presence of van der Waals contact with Leu 596 for PEPCK polymorphic site Ile/Val 629.** Models are of the lid-open conformation of the 5^′^-exon-excised mitochondrial form. Color codes as for Figures 6 and 7 except that Leu 596 is orange. Ile/Val 629 always makes van der Waals contact with Trp 595's β-CH_2_ group with some part of its side chain, but the part varies. **a**) allele SDI, no van der Waals contact between 596 and 629; **b**) allele SDV, strong van der Waals contact between 596 and 629. Small yellow numbers are distances between the indicated atoms in Å units.

Figures 5a and b also locate the polymorphic amino acid sites shared between species, which are illustrated in more detail in Figures 6, 7, and 8. Each can have different interactions with nearby invariant amino acids, depending on their own or other polymorph segregations, on catalytic stage as above, and on different compartment forms, e.g.:

• Gly/Ser 335 begins a very short loop connecting two β-strands, Gly 318 – Asp 334 and Val 338 – Ile 342. Gly^′^s ″side chain″ is one immobile proton, while Ser^′^s hydroxymethyl side chain is much larger, more polar, and can hydrogen-bond *via* its mobile hydroxyl proton. Either Gly or Ser can hydrogen-bond between its backbone nitrogen and the side-chain carboxyl of Asp 334; Ser′s hydroxyl can hydrogen-bond with the carboxyls of either Asp 334 or Asp 336. Bonding alternatives for Gly/Ser 335, tracking segregation of Ile/Val 629, are illustrated in Figure 6.

• Asp/Glu 503 is in α-helix Phe 501 – Ser 510. Asp and Glu differ in length of side chains (1 *vs.* 2 –CH_2_– groups), each ending in a carboxyl group. The guanidino side chain of nearby Arg 272 can form a salt bridge with the carboxyl of Asp/Glu 503, or hydrogen-bond to the backbone carbonyl group of Glu 503. Arg 506^′^s guanidino side chain, in the same α-helix as Asp/Glu 503, can also form a salt bridge with their carboxyl. These bonding possibilities, again tracking segregation of Ile/Val 629 but in the ″lid closed″ configuration of the cytosol form, are shown in Figure 7.

• Ile/Val 629 is near one end of the 3^′^ α-helix Asn 616 – Gln 630. Their nonpolar side chains, whose volumes differ by one –CH_2_– group, make van der Waals contact with Trp 595, and often Leu 596, in a different α-helix starting with Lys 592. In comparison to salt bridges or hydrogen bonds, van der Waals contacts occur over a wider range of carbon-carbon distances (hence different values of contact energy), which may be grouped: contact 2.95 – 4.5 Ǻ, marginal contact 4.5 – 5.2 Ǻ, no contact > 5.2 Ǻ cf. [33]. Figure 8 shows the absence or presence of van der Waals contact with Leu 596 for Ile 629 *vs.* Val 629, with the other polymorphic sites the same in each case.

Polymorphic variants may alter their nearby bonding contacts by changes of volume or polarity, as in absence/presence of a salt bridge to Arg 506 with the extension of 503^′^s side chain between alleles SDI and SEI (Figures 7a,c). Further, variants^′^ changes in properties can propagate their effects across PEPCK^′^s structure to alter distant bonding patterns, as, e.g., shown by responses of Ser 335 (Figures 6b,c) or Asp 503 (Figures 7a,b) bonding patterns to segregation of Ile/Val 629.

Additional file 5 lists all combinations of the eight allelic variants^′^ intramolecular bonding across catalytic stages and compartment forms. No two alleles display the same combination of bonds. Some additional suggestive patterns emerge from this file, for example:

• among the 48 cases, there is just one of side-chain polar bonds – hydrogen bond or salt bridge – occurring at both 335 and 503 sites in the same allele (GEV, in closed conformation of the full mitochondrial form), though four would be expected at random from the frequencies of those bonds′ occurrences. This might represent a mutual steric constraint.

• GDV and GDI alleles never show polar bonding between Asp 503 and Arg 272 in 12 cases, in contrast to the other 6 alleles which show 13 such bonds among 36 cases (exact binomial test [26] ×* = 244, P, 0.02). This may arise from the combination of Gly 335^′^s small volume and Asp 503^′^s short side chain, keeping Arg 272 and Asp 503 side chains distant from one another.

• the full mitochondrial form shows the fewest polar bonds by variants at site 335 (1/16) compared to the cytosol and the 5^′^-exon-excised mitochondrial forms (13/32; ×* = 2.47, P < 0.02).

• The cytosol form shows generally greater van der Waals contact distance between Ile/Val 629 and Leu 596 when closed than when open. This effect is less pronounced in the full mitochondrial form and is slightly reversed in the 5^′^-exon-excised mitochondrial form.

In summary, these results show that each allelic bond combination may make a different potential energy contribution to the stabilization of the protein^′^s structure as a whole, or of one part of the catalytic cycle (i.e. open or closed) *vs.* the other. By such effects, the alleles might alter either catalytic function or thermal stability, or both, of the PEPCK enzyme, and might do so differently among compartment-specific forms.

### Population-genetic testing of hypotheses for shared PEPCK polymorphisms

The species studied here, *Colias eurytheme* and *C. meadii*, represent in North America the lowland species complex and an alpine/northern species complex, respectively. They are fully reproductively isolated [34] and are well separated in phylogeny [35]. We now test population-genetic explanations for their sharing of 3 major (p_2_ ≥ 0.05) PEPCK polymorphisms, at codons 335, 503, and 629, and their close similarity of allele frequencies at codons 335 and 503 (above). Obvious alternative hypotheses are that some form of balancing selection maintains these polymorphisms in the two species, or instead that the variation is selectively neutral and subject to genetic drift.

We first consider the null hypothesis of selective neutrality. We assume the two *Colias* species diverged from an ancestral species *t* generations before the present. A non-synonymous polymorphism shared by the two species could result from a single mutation that occurred in the ancestral species and drifted to the observed frequencies in the descendant species. The probability of this approaches zero as t increases. It is also possible that independent mutations occurred in each descendant species after the split and drifted to the observed frequencies.

We have used the coalescent simulation program ″ms″ [36] to simulate this scenario with a symmetric two-allele mutation assumption, so that mutations can occur both in the ancestral species and in the two descendant species. The simulations are used to assess the probability, given the null hypothesis that the variants are drifting neutrally, that the variants' frequencies in the descendant species would be as similar as or more similar than their observed values. We denote the frequency of the second allele at a particular codon in species A (*C. eurytheme*) and B (*C. meadii*) by p_2A_ and p_2B_, respectively. From the ms program's output we estimate the probability that |p_2A_ - p_2B_| is less than or equal to the observed allele frequency difference. This probability will be a function of time since species A and B split from their common ancestor, scaled by effective population size: i.e., t/2N_e_. To choose a focal set of simulation outcomes from which to estimate this null probability, we set the condition that (p_2A_ + p_2B_) = observed value. This allows an unequivocal ordering of |p_2A_ – p_2B_| values from maximum to minimum agreement with the null hypothesis, hence minimum to maximum agreement with its selective alternative.

We estimate overall θ_ss_ = 4N_e_μ_ss_ [N_e_ = effective population size, μ_ss_ = synonymous (= neutral) mutation rate], a central parameter for these simulations, based on values of S_ss_, the number of segregating synonymous sites, from glycolytic genes of each *Colias* species: glyceraldehyde phosphate dehydrogenase GAPdH, phosphoglycerate kinase PGK, phosphoglycerate mutase PGAM (*C. eurytheme* only), pyruvate kinase PK, and triose phosphate isomerase TPI. These genes give no evidence of balancing or positive-directional selection on nonsynonymous sites, which if present could bias estimates by causing neutral variants to "hitchhike" on such sites (W. Watt *et al.*, unpublished). We correct estimates from sex-linked TPI for its N_e_ being *a priori* ¾ that of the other genes. The average value among estimates from these genes is θ_ss_ = 0.0556. We subdivide this following the common observation that transition mutants are twice as common as transversion mutants overall, and the fact that for any base pair one transition and two transversions are possible as single mutants. Thus, in our primary simulation analysis, to test transition polymorphisms we set θ_ss-tr_ = 0.037, and for transversion polymorphisms we set θ_ss-tv_ = 0.009.

We do not use a value of θ_ss_ from PEPCK itself in our primary analysis, as on the alternative hypothesis of selective maintenance of the shared polymorphisms, θ_ss_ might well be elevated (by hitchhiking) above neutral expectations, biasing any further calculations. However, a variant of the neutral null hypothesis would invoke a PEPCK-specific elevation of the mutation-rate component of θ_ss_ to explain high levels of PEPCK variation. We can, therefore, ask what is the probability of finding |p_2A_ - p_2B_| less than or equal to the observed allele frequency difference using PEPCK θ_ss_ in simulations, to see if elevated mutation rate could explain observed results *on a neutral assumption*. The overall θ_ss_ value averaged between *C. eurytheme* and *C. meadii* = 0.0892, so θ_ss-tr_ = 0.059 and θ_ss-tv_ = 0.015 for these additional simulations.

Next, what should be the “choice rule” for which PEPCK codons to test? Test power would be poor for small total numbers of the second allele at each codon, so our rule is that the total counts (n_2A_ + n_2B_) be ≥ 10 (out of 72, given 36 sequences for each species). Therefore, on one hand, besides the shared codons of primary interest, we should also test codon 11 of the mitochondrial PEPCK form, polymorphic for Arg/Lys. This has (n_2A_ + n_2B_) = 16, which satisfies the choice rule although it is polymorphic only in *C. eurytheme*, not *C. meadii*, as this is a possible outcome of the null hypothesis. But on the other hand, if the mitochondrial-targeting exon which includes codon 11 is indeed excised after PEPCK enters the mitochondrion (above), codon 11's polymorphism would not be expressed, would be synonymous in functional terms, and should not be tested with the other codons. Hence we report our significance testing both with and without inclusion of codon 11.

We ran 2 × 10^7^ simulations of 36 sampled sequences for each of two species and for each of the codon polymorphisms (codons 11, 335, and 629, transitions, and codon 503, transversion), filtered their output for cases satisfying [(p_2A_ + p_2B_) = observed value], and tabulated the fraction of those with (|p_2A_ – p_2B_| ≤ observed value) at intervals of t/2N_e_ between 0.2 and 2.6 as our null-hypothesis probability. Table 4 presents these results.

**Table 4:** Probabilities under genetic drift of observed or greater frequency similarity of PEPCK codon variants between species

				**Codons**			
	**θ**_**ss**_**from 5 genes**				**θ**_**ss**_**from PEPCK**		
	**11**	**335**	**503**	**629**	**335**	**503**	**629**
t/2N_e_							
0.2	1.0	0.340	0.221	0.675	0.342	0.234	0.679
0.4	″	0.215	0.092	0.431	0.210	0.097	0.430
0.6	″	0.144	0.047	0.275	0.142	0.049	0.286
0.8	″	0.073	0.032	0.188	0.091	0.036	0.205
1.0	″	0.059	0.021	0.138	0.061	0.027	0.154
1.2	″	0.043	0.016	0.101	0.043	0.016	0.123
1.4	″	0.028	0.013	0.083	0.029	0.014	0.103
1.6	″	0.020	0.011	0.070	0.024	0.015	0.092
1.8	″	0.018	0.011	0.067	0.019	0.014	0.086
2.0	″	0.014	0.008	0.059	0.016	0.014	0.082
2.2	″	0.012	0.007	0.056	0.015	0.013	0.083
2.4	"	0.011	0.007	0.052	0.012	0.012	0.077
2.6	"	0.009	0.006	0.052	0.012	0.012	0.072

Outcomes for values of species’ separation time (t) scaled by effective population size (2N_e_), calculated by simulation using “ms” software [36] as described in the text. Alternative sources of θ_ss_ for simulations are explained in the text. P values for codon 11 are the same for both θ_ss_ sources.

For comparison to Table 4, we estimate t/2N_e_ for our two *Colias* species *via* genetic statistics of synonymous (assumed neutral) variation at *Colias* GAPdH, hexokinase HK, PGK, PK, PGAM (both species), and TPI. We define these symbols: π_ssA_ or π_ssB_ = synonymous nucleotide diversity in species A or B; π_ssAB_ = between-species synonymous nucleotide diversity; π_ssCA_ = synonymous nucleotide diversity in the most recent common ancestor (MRCA); μ = mutation rate; N_e_ = effective population size; t = time in generations since MRCA; E(x) = expected value of x. Next, we assume π_ssA_ ~ π_ssB_ ~ π_ssCA_. π_ssA_ ~ π_ssB_ is evident for all 6 genes (W.B. Watt *et al.*, unpublished); that these values also reflect π_ssCA_ is reasonable, given present patterns of *Colias* speciation by differentiation of large "semispecies" populations without evident bottlenecking or founder effects [37,38]. Then:

(1) $$E\left(\pi_{\mathrm{ssAB}}\right)=2\mathrm{\mu t}+\pi_{\mathrm{ssCA}}$$

Assuming *π*_*ssCA*_ ∼ *π*_*ssA*_, *E*(*π*_*ssAB*_ - *π*_*ssA*_) = 2*μt*

(2) $$E\left(\pi_{\mathrm{ssA}}\right)=4N_{e}\mu$$

(3) $$E\left[\left(\pi_{\mathrm{ssAB}}-\pi_{\mathrm{ssA}}\right)/\pi_{\mathrm{ssA}}\right]≅2\mathrm{\mu t}/4N_{e}\mu =t/2N_{e}$$

By a parallel argument, *E*[(*π*_*ssAB*_ - *π*_*ssB*_)/*π*_*ssB*_ ≅ 2*μt*/4*N*_*e*_*μ* = *t*/2*N*_*e*_.

Estimates of t/2N_e_ were made for each species in turn at each of the 6 genes listed above, again correcting estimates from TPI for its smaller N_e_. The final average t/2N_e_ over all 6 genes and 2 species is 1.828 ± 0.635 (mean ± standard error of mean).

Table 5 applies “Fisher’s method” of combining probabilities [27] to the joint analysis of “ms” simulation results for the three polymorphic codons shared among species, with and without the mitochondrial codon 11 polymorphism as discussed above. We used t/2N_e_ = 1.80 as the closest tabulated value less than our averaged estimate. As Table 5 shows, using θ_ss_ estimated from the five genes as above, it is highly unlikely that these polymorphisms would be as or more similar than observed due to neutral drift, whether (P = 0.004) or not (P = 0.001) codon 11 is included in the analysis. This is so even though at codon 629 the polymorphism does differ significantly in frequency between species (above). Using θ_ss_ from PEPCK itself does not change these conclusions importantly: including codon 11 in the simulations, P = 0.006, while without codon 11 P = 0.002. Thus an elevated PEPCK-specific mutation rate combined with neutrality is also rejected as an explanatory hypothesis for the shared polymorphisms. These results support the alternative working hypothesis that codon polymorphisms 335, 503, and 629 are shared between species because they are maintained by parallel natural selection in the two species.

**Table 5:** **Probabilities, on null hypothesis, of frequency similarities at PEPCK polymorphic codons in two *Colias *species**

	**Amino acid polymorph**		**Probability calculations**			
	**counts**		**(t/2N**_**e**_ **= 1.8)**			
			**θ**_**ss**_**from 5 genes**		**θ**_**ss**_**from PEPCK**	
**Codon**	***C. eurytheme***	***C. meadii***	**P**	**ln P**	**P**	**ln P**
11	16 K, 20 R	36 K, 0 R	1.0	0.0	1.0	0.0
335	20 G, 16 S	15 G, 21 S	0.018	−4.02	0.019	−3.96
503	7 D, 29 E	4 D, 32 E	0.011	−4.51	0.014	−4.29
629	18 I, 18 V	6 I, 30 V	0.067	−2.70	0.086	−2.45
	*x*^2^ = − 2*Σ* ln *P*:		22.46		21.40	
	Probabilities of joint neutrality					
	including codon 11, df = 8		P = 0.004		P = 0.006	
	omitting codon 11, df = 6		P = 0.001		P = 0.002	

Site-specific probabilities estimated by simulation as described in the text and given in Table 4. t/2N_e_ estimate (actually 1.828) calculated as described in the text. Fisher’s method [27] is used to assemble joint probabilities of neutrality. Analysis using θ_ss_ from 5 genes tests basic neutral hypothesis *vs.* selective alternative; analysis using θ_ss_ from PEPCK tests whether an elevated mutation rate component of θ_ss_ could allow the null hypothesis to be sustained. See the text for details.

## Discussion

### PEPCK and PGI: different forms of chronically maintained polymorphism?

We have found that neutral drift is quite unlikely to explain the sharing of PEPCK’s amino acid polymorphisms between *Colias* species. Thus it makes sense that PEPCK, as a candidate for selectively maintained chronic polymorphism among species, shows very high levels of genetic variability comparable to those of *Colias*’ PGI gene, whose amino acid polymorphism is maintained widely across the genus by strong balancing selection [4,39,40].

However, the cases differ in detail. PGI polymorphism is maintained in *C. eurytheme* and *C. meadii* without preserving allelic identity between species. At PGI, a two-transversion change, Gly 370 GGG → Ser 370 TCG, was fixed by a selective sweep in the midst of chronic polymorphism at other codon sites [4], somewhere in phylogeny between more basal *C. meadii* and derived *C. eurytheme*[35]. This increases the *eurytheme* PGI genotypes’ thermal stabilities compared to those of *meadii*[41], fitting with differences in thermal ecology between the species. In contrast, PEPCK’s polymorphism engages the same 3 codons between species although, despite the inter-species similarity of amino acid variant frequencies at codons 335 and 503, the increase of p_2_ frequency at codon 629 from basal *C. meadii* to derived *C. eurytheme* does change several multicodon allele frequencies (Table 3).

The amino acid polymorphs of PEPCK and PGI have one structural feature in common: they occur outside their enzymes' catalytic centers. This is often so for natural variants that change catalytic (or stability) properties of enzymes without altering their reaction mechanisms [42]. But other structural aspects of these polymorphisms differ between the genes. PGI, with interpenetrated dimeric structure, is completely inactive as a dissociated monomer, so that no separable allelic properties exist beyond sequence differences themselves, and the genotype is the minimum unit of function and thus of performance or fitness effects. In contrast, PEPCK is active catalytically as a single polypeptide, so allelic structural and functional properties exist distinct from genotypic properties, which would be linear combinations of allelic ones. If PEPCK’s genotypes do differ in function, they could, for example, increase heterozygotes’ breadth of function across the range of a state variable such as temperature, or differ in balances of alternate metabolic roles (below).

Molecular evolutionists have long recognized the prevalence of conservation of sequence *via* stabilizing (“purifying”) selection across broad clades. In contrast, polymorphism is often seen as transient, whether neutral or selected, with “exceptions” recognized for recombination-suppressing inversion blocks as in *Drosophila*[43], or for frequency-dependent cases such as host-pathogen “arms races” [44-46] or self-incompatibility systems [47]. Cases such as PGI, phosphoglucomutase [48-50], and now probably PEPCK, demonstrate that chronic polymorphism may often be a long-term, stable response to multiple-scale environmental variation, albeit perhaps predisposed by complexities of protein structure (PGI) and/or metabolic role (PEPCK).

Accordingly, studies of the kinetic properties and thermal stabilities of the PEPCK variants will be of high priority, testing the present working hypothesis of selective maintenance for their polymorphism. If functional differences are indeed found, these may well give clues to the genotype-phenotype-environment interactions responsible for variants’ maintenance – as was the case for *Colias* PGI. Field studies to test those interactions will follow in turn.

### Compartmentation: functional rôles and expression strategies among taxa

The concept of “elementary flux modes” expresses how a group of enzyme steps can be deployed to execute different reaction series serving distinct metabolic functions – e.g., glycolysis *vs.* gluconeogenesis, or interactions of glycolysis with the pentose shunt [51]. As seen above, expression of PEPCK in both cytosol and mitochondria may support alternative elementary flux modes. Krebs cycle carbon skeletons derived from dietary or stored lipids or amino acids could be converted by mitochondrial PEPCK from OAA to PEP, then moved to the cytoplasm (by the tricarboxyl transporter [52]) for reverse-glycolytic support of glycerol synthesis or storage in glycogen [1]. Otherwise, cytosolic PEPCK could prime the Krebs cycle to match large transients in glycolytic flux (such as seen in insect flight), by diverting part of this flux from PEP into OAA, thence into mitochondria, perhaps *via* the malate shuttle, to react with acetyl-CoA derived from pyruvate [2].

How these flux modes might interact with functional effects of the *Colias* PEPCK amino acid polymorphisms remains to be explored. But in addition, the alternative strategies for PEPCK forms’ expression – splice variants in higher Lepidoptera *vs.* full-scale paralogous genes of independent origin in vertebrates and in Diptera – bespeak a level of adaptive specialization on a large scale. They may, for example, reflect deep clade differences in nutritional mass-energy budget structures. Thus multiple levels of evolutionary comparison are evoked by our present findings.

These expression strategy differences have implications for studies of clade structure itself. It was proposed that PEPCK sequences might offer good phylogenetic signal differentiating Mesozoic to early Cenozoic divergences of insect taxa, mainly in Lepidoptera but with other insects including *Drosophila* as outgroups [53]. The part of PEPCK studied is in the common block of splice-variant sequences in *Bombyx* and *Colias*, a region also quite similar between the *Drosophila* paralogs. However, what are the most appropriate outgroups, and whether basal Lepidoptera concur in the strategy of *Bombyx* and *Colias*, or display another expression pattern which may complicate sorting out sequence homology *vs.* paralogy, are open questions which systematists must address if they study this gene.

### PEPCK and the place of specific-gene studies in a time of genomic variation surveying

High-throughput sequencing and variation surveys using it have remarkable power to screen genomes for genetic evidence of evolution [54]. But it is increasingly recognized that “genomics is not enough” to overcome underdetermination of genetic variation patterns by theoretically possible alternative processes [55]. (This problem should be clear even from simple population genetics: e.g., a heterozygote deficiency compared to Hardy-Weinberg expectation may arise from inbreeding *or* a Wahlund effect *or* underdominance in fitness, and only study of process can choose the right explanation.) Genome-wide surveys can at best evoke working hypotheses to be tested by study of varying mechanisms in specific gene systems [56-58]. The focus of our molecular survey on a central, functionally well-known pathway has allowed augmentation of PEPCK’s genetic statistics with initial structural and population-genetic studies, and poises the case for mechanistic testing. This study of *Colias* PEPCK, like our earlier work [39], engages diverse processes which can shape natural variation, from protein-specific structural predispositions or constraints to epistatic interaction among nearby enzyme steps, systemic pathway organization and enzymes’ roles in it, or “global” issues of energy allocation or network connectedness. Case studies of natural variation’s effects are a potent source of insight into partition of evolutionary causes among these processes.

This does not imply a surrender to evolutionary particularism. On the contrary, we seek, with Whitehead [59], “…to see the forest by means of the trees”. The now-obvious universality of the genetic code, the “unity of biochemistry”, and other unifying concepts of molecular and physiological evolution were established by detailed studies of the molecular mechanisms of diverse organisms – in complement to the distillation of natural selection and other early evolutionary generalities out of many specific cases by Darwin and his successors. In biology, the path to heuristic generalization runs through the comparative study of specificity [42,60].

This situation underscores the importance of a difference of evolutionary paradigms: an approach which is self-limited to amechanistic pattern analysis in evolution [61], *vs.* a view which values patterns as starting points but, as in our earlier work and as begun here, tests their causes by mechanistic studies of genotype-phenotype-environment interactions [62-64] which are the actual drivers of natural selection [65]. Increasing focus on the power of the latter paradigm [66,67] will lead to deeper insight into evolutionary processes and into realistic generalities concerning them.

## Conclusions

We've pursued diverse approaches to PEPCK’s evolution in two *Colias* species:

• phylogenetic comparisons of strategy for expression of cell compartment forms, finding splice variation in *Colias* (like *Bombyx*) as contrasted to paralogous gene divergence in other clades;

• finding extensive genetic variation at nucleotide and amino acid levels, including three amino acid polymorphisms which are shared among species, in two cases with similar frequencies;

• homology-based modelling, finding that these three polymorphisms may have both local structural impacts and longer-range interactions among their distinct locations in PEPCK structure;

• population genetic simulation, testing the null hypothesis of neutrality of amino acid polymorphs and finding it improbable (0.001 ≤ P ≤ 0.006), leaving the alternative of natural-selective maintenance.

Each by itself gives important clues to causes of the gene’s extensive variation in context of the splice-based evolutionary strategy of compartment-specific PEPCK expression, in contrast to the paralogy seen in *Drosophila* and in vertebrates. Together they offer a coherent hypothesis of selectively maintained polymorphism, chronically persistent among species. This hypothesis is now poised for further test, clarifying PEPCK’s genotype-phenotype-environment interaction by studies of PEPCK’s allelic and genotypic functions, performances, and fitnesses.

## Abbreviations

ATP: Adenosine triphosphate; bp: Base pair(s); cDNA: Coding DNA; GAPdH: Glyceraldehyde phosphate dehydrogenase; GTP: Guanosine triphosphate; HK: Hexokinase; kb: Kilobase(s); MRCA: Most recent common ancestor; mRNA: Messenger RNA; μ: Mutation rate; N_e_: Effective population size; nss: Nonsynonymous (nt) sites; nt: Nucleotide; OAA: Oxaloacetic acid; P: Probability of a statistical outcome being due to chance; p_2_: Frequncy of second allele at a gene or codon; PCR: Polymerase chain reaction; PDB: Protein Data Bank; PEP: Phosphoenolpyruvate; PEPCK: Phosphoenolpyruvate carboxykinase; PGAM: Phosphoglycerate mutase; PGI: Phosphoglucose isomerase; PGK: Phosphoglycerate kinase; PK: Pyruvate kinase; π: Nucleotide diversity; S: Segregating sites; ss: Synonymous (nt) sites; Standard one-letter abbreviations for amino acids; Standard one-letter abbreviations for nucleotide bases; Standard three-letter abbreviations for amino acids; Σ: Sum or total; t/2N_e_: Time of separation of species scaled by effective population size(s); θ: 4N_e_μ; TPI: Triose phosphate isomerase; UTR: Untranslated region; x*: Test parameter (normal deviate) for exact binomial test.

## Competing interests

The authors declare that they have no competing interests.

## Authors’ contributions

WBW conceived and organized the study. BW and WBW collected specimens. BW, EW, and WBW collected and analyzed sequence data. WBW carried out structural studies. RRH developed the algorithm for estimating t/2N_e,_ and designed and ran the simulations with input from WBW. WBW wrote the paper with input from other authors, all of whom read and approved the final manuscript.

## Supplementary Material

Additional file 1 **Amplifying and sequencing primers for study of *Colias *PEPCK.** Amplifying primers are of course used for sequencing as well. ″5end″ and ″3end″ primers are located in 5^′^- and 3^′^-untranslated regions (UTRs). S and A denote sense and antisense primer directions, respectively. Overlapping gene subsets amplified for sequencing: MVY-S-5end and MLH-S2-5end / A1005 (one each per individual); S511C / A1519C; and S1487 / A3end. Primer numbers denote primers’ 3^′^-terminal nucleotide positions in the gene. Click here for file

Additional file 2 Reference sequences of PEPCK (GTP; EC 4.1.1.32) from diverse taxa and bioinformatics sources. Click here for file

Additional file 3 **Tests of linkage disequilibrium among varying amino acid sites.** Abbreviations: amino acids, standard one-letter codes; obs, observed; exp, expected; D, linkage disequilibrium = pX_1_pX_4_ – pX_2_pX_3_; G_Yates(1)_, G statistic with Yates’ correction and 1 degree of freedom; Fisher’s exact, test of that name [ref]; P, probability of this or more extreme result by chance alone. a) tests of sites polymorphic in both *Colias* species. Site variant frequencies in main text Table 2. Correction of significance threshold for multiple tests by Dunn-Sidak method [ref], 6 tests, α^′^ = 0.0085. No linkage disequilibria are significant. b) tests of Arg/Lys 11, polymorphic only in mitochondrial 5^′^ exon of *C. eurytheme*, and the other polymorphic sites in that species. Site frequencies at site 11: Arg 0.556, Lys 0.444; other site frequencies as above. 3 tests, α^′^ = 0.017. No linkage disequilibria are significant. Click here for file

Additional file 4 **Alignment of *Rattus *PEPCK (PDB 2qew) with *Colias *PEPCK (allele GDV) as amino acid sequences.** Alignment generated with Modeller [19]. “*” denotes identity of residue between sequences. Click here for file

Additional file 5 **Absence/presence of polymorphic amino acid variable bonds with sidechains of nearby invariant amino acids in *Colias *PEPCK.** Abbreviations: H bond, hydrogen bond; Ǻ, Ǻngstrom unit of length; vdW, van der Waals; marg, marginal. Alleles identified by one-letter amino acid codes at each of the three polymorphic sites shared between species. Invariant amino acid sites bonding to variable amino acids are identified by number prior to bond length in Ǻ. 272-503 H bonds engage the backbone carbonyl of amino acid 503. All bond types, including distance criteria for marginal or absent van der Waals contacts, are discussed in the main text and many are illustrated in Figures 6, 7, 8. Click here for file
