# Supplementary material for: A genetic polymorphism evolving in parallel in two cell compartments and in two clades
Source: BMC Evol Biol. 2013 Jan 12;13:9. doi: 10.1186/1471-2148-13-9 (PMC3556304; doi:10.1186/1471-2148-13-9)
Supplement: Additional file 1 — Amplifying and sequencing primers for study of Colias PEPCK. Amplifying primers are of course used for sequencing as well. ″5end″ and ″3end″ primers are located in 5′- and 3′-untranslated regions (UTRs). S and A denote sense and antisense primer directions, respectively. Overlapping gene subsets amplified for sequencing: MVY-S-5end and MLH-S2-5end / A1005 (one each per individual); S511C / A1519C; and S1487 / A3end. Primer numbers denote primers’ 3′-terminal nucleotide positions in the gene. [file 1471-2148-13-9-S1.docx]

Name 5'→3' sequence (and comments/variants)

PCK-MVY-S5end CGTGAATAACATAAAATAAGCAGAG

(Mitochondrial 5'-UTR )

PCK-MLH-S25end TTTTTWATTTATTTTGCGTCC

(Cytosolic 5'-UTR)

PCK-A491C CATAGAGAATGGGATCACGTACA

PCK-S511C CCATGTACGTGATCCCATTCTCTA

PCK-S983 CTACATAGCAGCCGCTTTTCC

PCK-A1005C TTGGGGTCATCATCGCRAGG

PCK-S1487 TGCTGGTAAAGTAGTAATGCACGA

PCK-A1519C GTAMTCCCCGAAGTTGTAGCC

PCK-S1840 CGATAGGAAAATACTTCAAAGAGG

PCK-A3end GGTYTATBAWGTBKTTWGGTAWRTABAATTTATC

**Additional File 1. Amplifying and sequencing primers for study of *Colias* PEPCK.**

Amplifying primers are of course used for sequencing as well. "5end" and "3end" primers are located in 5'- and 3'-untranslated regions (UTRs). S and A denote sense and antisense primer directions, respectively. Overlapping gene subsets amplified for sequencing: MVY-S-5end and MLH-S2-5end / A1005 (one each per individual); S511C / A1519C ; and S1487 / A3end. Primer numbers denote primers’ 3′-terminal nucleotide positions in the gene.
