# Supplementary material for: A genetic polymorphism evolving in parallel in two cell compartments and in two clades
Source: BMC Evol Biol. 2013 Jan 12;13:9. doi: 10.1186/1471-2148-13-9 (PMC3556304; doi:10.1186/1471-2148-13-9)
Supplement: Additional file 2 — Reference sequences of PEPCK (GTP; EC 4.1.1.32) from diverse taxa and bioinformatics sources. [file 1471-2148-13-9-S2.docx]

Taxon Compartment/form* Source Sequence type Accession or other reference number

*Bombyx mori* Cytosol/1 ButterflyBase EST lib (mRNA) BMP026541_1

*Bombyx mori* Cytosol/2 ButterflyBase EST lib (mRNA) BMP026778_1

*Bombyx mori* Mitochondrion ButterflyBase EST lib (mRNA) BMP000843_1

*Bombyx mori* Cytosol/1 SilkDB Genomic DNA nscaf2789: 1157957-1172461 (minus strand)

*Bombyx mori* Cytosol/2 SilkDB Genomic DNA nscaf2789: 1180525-1192791 (minus strand)

*Bombyx mori* Mitochondrial 5′ exon SilkDB Genomic DNA nscaf2789: 1207835-~1207900 (minus strand)

*Bos taurus* Cytosol GenBank mRNA NM174737

*Bos taurus* Mitochondrion GenBank mRNA XM583200

*Colias* spp. Cytosol and

mitochondrion GenBank mRNA KC404923-KC404958 (cytosolic form),

KC409319-KC409354 (mitochondrial form)

*Danio rerio* Cytosol GenBank mRNA NM214751

*Danio rerio* Mitochondrion GenBank mRNA NM213192

*Drosophila melanogaster* Cytosol FlyBase mRNA CG17725

*Drosophila melanogaster* Mitochondrion FlyBase mRNA CG10924

*Gallus gallus* Cytosol GenBank mRNA M14229

*Gallus gallus* Mitochondrion GenBank mRNA J05419

*Homo sapiens* Cytosol GenBank mRNA NM002591

*Homo sapiens* Mitochondrion GenBank mRNA NM004563

*Mus musculus* Cytosol GenBank mRNA BC037629

*Mus musculus* Mitochondrion GenBank mRNA BC010318

*Mycobacterium* -- GenBank Genomic DNA AF332191

*Sulfolobus* -- GenBank Protein YP002841481

**Additional File 2. Reference sequences of PEPCK (GTP; EC 4.1.1.32) from diverse taxa and bioinformatics sources.**
