# Supplementary material for: A genetic polymorphism evolving in parallel in two cell compartments and in two clades
Source: BMC Evol Biol. 2013 Jan 12;13:9. doi: 10.1186/1471-2148-13-9 (PMC3556304; doi:10.1186/1471-2148-13-9)
Supplement: Additional file 3 — Tests of linkage disequilibrium among varying amino acid sites. Abbreviations: amino acids, standard one-letter codes; obs, observed; exp, expected; D, linkage disequilibrium = pX1pX4 – pX2pX3; GYates(1), G statistic with Yates’ correction and 1 degree of freedom; Fisher’s exact, test of that name [ref]; P, probability of this or more extreme result by chance alone. a) tests of sites polymorphic in both Colias species. Site variant frequencies in main text Table 2. Correction of significance threshold for multiple tests by Dunn-Sidak method [ref], 6 tests, α′ = 0.0085. No linkage disequilibria are significant. b) tests of Arg/Lys 11, polymorphic only in mitochondrial 5′ exon of C. eurytheme, and the other polymorphic sites in that species. Site frequencies at site 11: Arg 0.556, Lys 0.444; other site frequencies as above. 3 tests, α′ = 0.017. No linkage disequilibria are significant. [file 1471-2148-13-9-S3.docx]

a)

*C. eurytheme C.meadii*

obs exp obs exp

Amino acid sites Gamete X_i_ count pX_i_ pX_i_ count pX_i_ pX_i_

335 – 503 X_1_ GD 4 0.1111 0.1079 1 0.0278 0.0463

X_2_ GE 16 0.4444 0.4481 14 0.3889 0.3704

X_3_ SD 3 0.0833 0.0861 3 0.0833 0.0647

X_4_ SE 13 0.3611 0.3579 18 0.5000 0.5183

-- • --

D: +0.0031 -0.0185

G_Yates(1)_: 0.108, P = 0.74 0.032, P = 0.86

Fisher’s exact: P = 1.00 P = 0.63

------------------------------------------------

*C. eurytheme C.meadii*

obs exp obs exp

Amino acid sites Gamete X_i_ count pX_i_ pX_i_ count pX_i_ pX_i_

335 – 629 X_1_ GI 7 0.1944 0.2778 2 0.0556 0.0694

X_2_ GV 13 0.3611 0.2778 13 0.3611 0.3472

X_3_ SI 12 0.3333 0.2222 4 0.1111 0.0972

X_4_ SV 4 0.1111 0.2222 17 0.4722 0.4861

-- • --

D: -0.0988 -0.0138

G_Yates(1)_: 4.32, P = 0.038 <0.001, P = 0.997

Fisher’s exact: P = 0.023 P = 1.00

------------------------------------------------

*C. eurytheme C.meadii*

obs exp obs exp

Amino acid sites Gamete X_i_ count pX_i_ pX_i_ count pX_i_ pX_i_

503 – 629 X_1_ DI 2 0.0556 0.0972 1 0.0278 0.0185

X_2_ DV 5 0.1389 0.0972 3 0.0833 0.0926

X_3_ EI 16 0.4444 0.4028 5 0.1389 0.1482

X_4_ EV 13 0.3611 0.4028 27 0.7500 0.7407

-- • --

D: -0.0417 +0.0093

G_Yates(1)_: 0.718, P = 0.40 0.060, P = 0.81

Fisher’s exact: P = 0.40 P = 1.00

------------------------------------------------

b)

obs exp

Amino acid sites Gamete X_i_ count pX_i_ pX_i_

11 – 335 X_1_ RS 7 0.1944 0.2468

X_2_ RG 13 0.3611 0.3091

X_3_ KS 9 0.2500 0.1971

X_4_ KG 7 0.1944 0.2468

-- • --

D: -0.0523

G_Yates(1)_: 0.881, P = 0.348

Fisher’s exact: P = 0.313

------------------------------------------------

obs exp

Amino acid sites Gamete X_i_ count pX_i_ pX_i_

11 – 503 X_1_ RE 17 0.4722 0.4481

X_2_ RD 3 0.0833 0.1079

X_3_ KE 12 0.3333 0.3579

X_4_ KD 4 0.1111 0.0861

-- • --

D: +0.0247

G_Yates(1)_: 0.108, P = 0.74

Fisher’s exact: P = 0.675

------------------------------------------------

obs exp

Amino acid sites Gamete X_i_ count pX_i_ pX_i_

11 – 629 X_1_ RI 13 0.3611 0.2780

X_2_ RV 7 0.1944 0.2780

X_3_ KI 5 0.1389 0.2220

X_4_ KV 11 0.3056 0.2220

-- • --

D: +0.083

G_Yates(1)_: 2.852, P = 0.091

Fisher’s exact: P = 0.092

------------------------------------------------

**Additional File 3. Tests of linkage disequilibrium among varying amino acid sites.**

Abbreviations: amino acids, standard one-letter codes; obs, observed; exp, expected; D, linkage disequilibrium = pX_1_pX_4_ – pX_2_pX_3_; G_Yates(1)_, G statistic with Yates’ correction and 1 degree of freedom; Fisher’s exact, test of that name [ref]; P, probability of this or more extreme result by chance alone. **a) tests of sites polymorphic in both *Colias* species**. Site variant frequencies in main text Table 2. Correction of significance threshold for multiple tests by Dunn-Sidak method [ref], 6 tests, α′ = 0.0085. No linkage disequilibria are significant. **b) tests of Arg/Lys 11, polymorphic only in mitochondrial 5′ exon of *C. eurytheme*, and the other polymorphic sites in that species.** Site frequencies at site 11: Arg 0.556, Lys 0.444; other site frequencies as above. 3 tests, α′ = 0.017. No linkage disequilibria are significant.
