# Supplementary material for: A genetic polymorphism evolving in parallel in two cell compartments and in two clades
Source: BMC Evol Biol. 2013 Jan 12;13:9. doi: 10.1186/1471-2148-13-9 (PMC3556304; doi:10.1186/1471-2148-13-9)
Supplement: Additional file 4 — Alignment of Rattus PEPCK (PDB 2qew) with Colias PEPCK (allele GDV) as amino acid sequences. Alignment generated with Modeller [19]. “*” denotes identity of residue between sequences. [file 1471-2148-13-9-S4.docx]

position 10 20 30 40 50 60

*Rattus* -----PQLHN-----GLDFS-A---KVIQG-----SLDSLPQEVRKFVEGNAQLCQPEYIH

*Colias* MVYFITRISKKFAQVALNCSRAAHQTALRGNKLSPQIAALTPKVRAFIERSAALCEPEHVH

identical * * * * * ** * * * ** ** *

position 70 80 90 100 110 120

*Rattus* ICDGSEEEYGRLLAHMQEEGVIRKLKKYDNCWLALTDPRDVARIESKTVIITQEQRDTVPI

*Colias* VCDGSEAEASALLQLMQNQGTLKPLPKYDNCWLARTDPADVARVESRTFICSERERDVVPA

identical ***** * ** ** * * ******** *** **** ** * * ** **

position 130 140 150 160 170 180

*Rattus* PKSGQ-SQLGRWMSEEDFEKAFNARFPGCMKGRTMYVIPFSMGPLGSPLAKIGIELTDSPY

*Colias* ARAGQKSALGNYIAPNDYDKAVTERFPGCMRGRTMYVIPFSMGPVGSPLSKIGVEITDSPY

identical ** * ** * ** ****** ************* **** *** * *****

position 190 200 210 220 230 240

*Rattus* VVASMRIMTRMGTSVLEALG-DGEFIKCLHSVGCPLPLKKPLVNNWACNPELTLIAHLPDR

*Colias* VVYSMRVMTRIGASVLEALRKDEHFVRCLHSVGR---SQTPGTAGWPCDPARTIILHRPAD

identical ** *** *** * ****** * * ****** * * * * * * * *

position 250 260 270 280 290 300

*Rattus* REIISFGSGYGGNSLLGKKCFALRIASRLAKEEGWLAEHMLILGITNPEGKKKYLAAAFPS

*Colias* SEIVSYGSGYGGNSLLGKKCFALRLGSVLARREGWLAEHMLIVGITDPRGRKRYIAAAFPS

identical ** * ****************** * ** ********** *** * * * * ******

position 310 320 330 340 350 360

*Rattus* ACGKTNLAMMNPTLPGWKVECVGDDIAWMKFDAQGNLRAINPENGFFGVAPGTSVKTNPNA

*Colias* ACGKTNLAMMTPSLPGYKVECVGDDIAWMKFDGDGVLRAINPENGFFGVAPGTSSSTNPIA

identical ********** * *** *************** * ****************** *** *

position 370 380 390 400 410 420

*Rattus* IKTIQKNTIFTNVAETSDGGVYWEGIDEPLAPGVTITSWKNKEWRPQDEEPCAHPNSRFCT

*Colias* MASVFKNTVFTNVAETKDGGVWWEGMG-T-APE-GLTDWKGKQWD--RKAPAAHPNSRFCT

identical *** ******* **** *** ** * ** * * * *********

position 430 440 450 460 470 480

*Rattus* PASQCPIIDPAWESPEGVPIEGIIFGGRRPAGVPLVYEALSWQHGVFVGAAMRSEA-----

*Colias* PAENCPIIDSEWESAAGVPISAILLGGRRPEGVPLVVEARDWAHGVFMGASMRSEATAAAE

identical ** ***** *** **** * ***** ***** ** * **** ** *****

position 490 500 510 520 530 540

*Rattus* --GKVIMHDPFAMRPFFGYNFGKYLAHWLSMAHRPAAKLPKIFHVNWFRKDKNGKFLWPGF

*Colias* HAGKVVMHDPFAMRPFFGYNFGDYLRHWLSMPQ-AGRQMPKIFHVNWFRKDSEGKFLWPGF

identical *** **************** ** ***** ************ ********

position 550 560 570 580 590 600 610

*Rattus* GENSRVLEWMFGRIEGEDSAKLTPIGYVPKEDALNLKGLGDVNVEELFGISKEFWEKEVEE

*Colias* GENSRVLDWVLRRCEEEDCHVETPLGYIPKENALNTKDLGNINMKELFSIPKEFWLQEADA

identical ******* * * * ** ** ** *** *** * ** * *** * **** *

position 620 630 640

*Rattus* IDKYLEDQVNADLPYEIERELRALKQRISQM

*Colias* IGKYFKEEVGEDLPNEMWDQLMTLKKNVQNS

identical * ** * *** * * **

**Additional File 4. Alignment of *Rattus* PEPCK (PDB 2qew) with *Colias* PEPCK (allele GDV) as amino acid sequences.** Alignment generated with Modeller [19]. “*” denotes identity of residue between sequences.
