# Supplementary material for: A genetic polymorphism evolving in parallel in two cell compartments and in two clades
Source: BMC Evol Biol. 2013 Jan 12;13:9. doi: 10.1186/1471-2148-13-9 (PMC3556304; doi:10.1186/1471-2148-13-9)
Supplement: Additional file 5 — Absence/presence of polymorphic amino acid variable bonds with sidechains of nearby invariant amino acids in Colias PEPCK. Abbreviations: H bond, hydrogen bond; Ǻ, Ǻngstrom unit of length; vdW, van der Waals; marg, marginal. Alleles identified by one-letter amino acid codes at each of the three polymorphic sites shared between species. Invariant amino acid sites bonding to variable amino acids are identified by number prior to bond length in Ǻ. 272-503 H bonds engage the backbone carbonyl of amino acid 503. All bond types, including distance criteria for marginal or absent van der Waals contacts, are discussed in the main text and many are illustrated in Figures 6, 7, 8. [file 1471-2148-13-9-S5.docx]

Codons

335 503 629

form allele closed open closed open closed open

cyto GDV H bond H bond no bond salt bridge vdW contact vdW contact

334 2.61 Ǻ 334 2.61 Ǻ 506 3.19 Ǻ 596 2.95 Ǻ 596 2.95 Ǻ

GDI H bond no bond no bond no bond vdW contact vdW contact

334 2.61 Ǻ 596 3.19 Ǻ 596 2.95 Ǻ

GEV H bond no bond no bond salt bridge no contact vdW contact

334 2.61 Ǻ 272 3.04 Ǻ 596 6.52 Ǻ 596 3.41 Ǻ

GEI no bond H bond no bond no bond vdW contact vdW contact

334 2.61 Ǻ 596 3.73 Ǻ 596 2.96 Ǻ

SDV no bond no bond salt bridge no bond vdW contact vdW contact

272 2.89 Ǻ 596 3.65 Ǻ 596 3.29 Ǻ

SDI no bond no bond no bond salt bridge marg contact marg contact

272 2.71 Ǻ 596 4.96 Ǻ 596 4.97 Ǻ

SEV no bond no bond no bond salt bridge vdW contact vdW contact

272 2.95 Ǻ 596 3.07 Ǻ 596 2.95 Ǻ

SEI no bond H bonds: 334 2.61 Ǻ salt bridge no bond marg contact vdW contact

Ser –OH 334, 336 506 3.20 Ǻ 596 4.96 Ǻ 596 2.95 Ǻ

3.11 Ǻ, 3.30 Ǻ

Additional File 5 continued…

mito GDV no bond no bond no bond no bond vdW contact vdW contact

596 3.45 Ǻ 596 3.05 Ǻ

GDI no bond no bond no bond no bond marg contact no contact

334 2.61 Ǻ 596 5.06 Ǻ 596 6.10 Ǻ

GEV H bond no bond salt bridge no bond no contact vdW contact

334 2.61 Ǻ 272 2.83 Ǻ 596 6.50 Ǻ 596 3.10 Ǻ

GEI no bond no bond H bond no bond vdW contact vdW contact

272 2.66 Ǻ 596 2.95 Ǻ 596 2.95 Ǻ

SDV no bond no bond salt bridge no bond vdW contact vdW contact

272 2.79 Ǻ 596 3.04 Ǻ 596 3.38 Ǻ

SDI no bond no bond no bond no bond vdW contact vdW contact

272 2.71 Ǻ 596 3.60 Ǻ 596 2.95 Ǻ

SEV no bond no bond H bond no bond vdW contact vdW contact

272 2.66 Ǻ 596 2.95 Ǻ 596 3.42 Ǻ

SEI no bond no bond salt bridge salt bridge no contact marg contact

506 3.20 Ǻ 272 2.89 Ǻ 596 6.41 Ǻ 596 4.86 Ǻ

mito-excised GDV no bond no bond no bond no bond vdW contact vdW contact

596 3.41 Ǻ 596 3.62 Ǻ

GDI H bond H bond no bond no bond vdW contact vdW contact

334 2.61 Ǻ 334 2.61 Ǻ 596 4.37 Ǻ 596 2.95 Ǻ

Additional File 5 continued…

GEV H bond no bond no bond salt bridge vdW contact vdW contact

334 2.61 Ǻ 272 2.80 Ǻ 596 3.46 Ǻ 596 3.18 Ǻ

GEI no bond no bond no bond salt bridge vdW contact vdW contact

272 3.27 Ǻ 596 3.18 Ǻ 596 4.42 Ǻ

SDV H bond no bond no bond no bond vdW contact vdW contact

334 2.61 Ǻ 596 3.97 Ǻ 596 3.68 Ǻ

SDI H bond no bond no bond no bond vdW contact no contact

334 2.61 Ǻ 596 2.95 Ǻ 596 6.08 Ǻ

SEV no bond no bond salt bridge salt bridges 272 vdW contact vdW contact

506 3.22Ǻ 2.70 Ǻ, 506 2.72 Ǻ 596 3.30 Ǻ 596 2.95 Ǻ

SEI H bonds: 334 no bond no bond salt bridge vdW contact vdW contact

2.61 Ǻ, Ser 272 2.81 Ǻ 596 2.95 Ǻ 596 3.88 Ǻ

–OH 334, 336

3.11 Ǻ, 3.30 Ǻ

**Additional File 5. Absence/presence of polymorphic amino acid variable bonds with sidechains of nearby invariant amino acids in *Colias* PEPCK.** Abbreviations: H bond, hydrogen bond; Ǻ, Ǻngstrom unit of length; vdW, van der Waals; marg, marginal. Alleles identified by one-letter amino acid codes at each of the three polymorphic sites shared between species. Invariant amino acid sites bonding to variable amino acids are identified by number prior to bond length in Ǻ. 272-503 H bonds engage the backbone carbonyl of amino acid 503. All bond types, including distance criteria for marginal or absent van der Waals contacts, are discussed in the main text and many are illustrated in Figures 6-8.
